# Supplementary material for: Structure-based design of covalent nanobody binders for a thermostable green fluorescence protein: Engineering covalent nanobody-GFP complexes
Source: Acta Biochim Biophys Sin (Shanghai). 2024 Dec 24;57(8):1363–70. doi: 10.3724/abbs.2024233 (PMC12368520; doi:10.3724/abbs.2024233)
Supplement: Highlight [file Highlight.docx]

**Highlight**

The green fluorescent protein (GFP) has revolutionized life sciences, illuminating countless biological processes. An ultra-thermostable GFP (TGP) offers enhanced stability over traditional jellyfish-derived GFP but lacks the extensive toolset, including nanobodies, available for GFP. Here, we present the crystal structure of TGP bound to a synthetic nanobody, revealing an atypical binding mode at atomic resolution. This detailed interaction has enabled the rational design of disulfide-linked TGP-nanobody complexes, one of which exhibits exceptional resistance to reducing agents. Our work broadens the toolkit for TGP and promotes its application in biological research, including high-purity purification of TGP-fusion proteins.

- Crystal structure reveals an atypical binding mode of a thermostable GFP-targeting nanobody
- Rational design of two disulfide-linked nanobody-TGP complexes
- A highly reducing agent-resistant disulfide complex offers new potential applications in biological research.
